# Photocatalyst Based on Nanostructured TiO_2_ with Improved Photocatalytic and Antibacterial Properties

**DOI:** 10.3390/ma16247509

**Published:** 2023-12-05

**Authors:** Roberta Irodia, Camelia Ungureanu, Veronica Sătulu, Vasilica Mihaela Mîndroiu

**Affiliations:** 1 Faculty of Chemical Engineering and Biotechnologies, National University of Science and Technology Politehnica Bucharest, 1-7 Polizu, 011061 Bucharest, Romania; roberta.irodia@upb.ro (R.I.); camelia.ungureanu@upb.ro (C.U.); 2 National Institute for Laser, Plasma and Radiation Physics, Atomiștilor 409, 077125 Măgurele, Romania; veronica.satulu@inflpr.ro

**Keywords:** nanostructures, blue-TiO_2_ nanotubes, antimicrobial, antibiotics, photocatalytic

## Abstract

This study shows an easy way to use electrochemistry and plasma layering to make Cobalt-Blue-TiO_2_ nanotubes that are better at catalysing reactions. Once a titanium plate has been anodized, certain steps are taken to make oxygen vacancies appear inside the TiO_2_ nanostructures. To find out how the Co deposition method changed the final catalyst’s properties, it was put through electrochemical tests (to find the charge transfer resistance and flat band potential) and optical tests (to find the band gap and Urbach energy). The catalysts were also described in terms of their shape, ability to stick to surfaces, and ability to inhibit bacteria. When Cobalt was electrochemically deposited to Blue-TiO_2_ nanotubes, a film with star-shaped structures was made that was hydrophilic and antibacterial. The band gap energy went down from 3.04 eV to 2.88 eV and the Urbach energy went up from 1.171 eV to 3.836 eV using this electrochemical deposition method. Also, photodegradation tests with artificial doxycycline (DOX) water were carried out to see how useful the study results would be in real life. These extra experiments were meant to show how the research results could be used in real life and what benefits they might have. For the bacterial tests, both gram-positive and gram-negative bacteria were used, and BT/Co-E showed the best response. Additionally, photodegradation and photoelectrodegradation experiments using artificial doxycycline (DOX) water were conducted to determine the practical relevance of the research findings. The synergistic combination of light and applied potential leads to 70% DOX degradation after 60 min of BT/Co-E irradiation.

## 1. Introduction

There has been a global rise in the consumption of various substances such as vitamins, immune boosters, combination medications, and cocktails of antivirals, antibiotics, steroids, and antifungals. However, antibiotics and dyes have emerged as a significant cause for concern [1,2,3,4]. Several species, including Salmonella and Pseudomonas aeruginosa, have exhibited resistance to tetracyclines (TCs) [5,6], which are the second most utilized family of antibiotics in both human and veterinary medicine. This resistance has emerged due to the efficient elimination of tetracycline through glomerular filtration in urine. Doxycycline (DOX) is a semi-synthetic tetracycline antibiotic that has a prolonged effect and is effective against a wide range of bacteria [7,8]. As the literature reported [9,10,11], doxycycline has a significant role in the COVID-19 therapy also.

*Salmonella*, a bacterial pathogen frequently associated with instances of foodborne diseases, has the potential to be present in water and wastewater systems in the event of fecal contamination. The treatment of leftover water to effectively eradicate microorganisms is of utmost importance. It is important to acknowledge that Salmonella could persist in water for a certain duration, especially in higher temperatures. However, its ability to multiply is often dependent on the availability of a nutrition source [12,13]. Pseudomonas aeruginosa, a prevalent bacteria commonly detected in aquatic environments and terrestrial habitats [14], can pose significant concerns in residual waters or wastewater. It is known for its ability to survive in various environmental conditions and persist in water for extended periods [15,16,17]. Treating residual waters to eliminate *Pseudomonas aeruginosa* is difficult due to its resistance to many disinfection methods, including some antibiotics [18,19]. Therefore, it is crucial to improve technological solutions for the elimination of organic substances and germs from water sources.

Consequently, the rise in water contamination has heightened the significance of electrochemical technologies encompassing chemical, physical, and biological processes. One illustration can be observed in the realm of water depollution, where photocatalysis techniques utilizing TiO_2_ nanostructures are employed. Titanium dioxide (TiO_2_) a nontoxic material, due to its great chemical stability, high resistance to photo corrosion, and inexpensive cost, has been one of the most investigated materials in the past few years [20,21]. TiO_2_ photocatalysts that are efficient and stable can be developed out of nanomaterials with morphologies ranging from 0D to 3D [22] and among all these forms, TiO_2_ nanotubes (NTs) are a part of 1D titanium nanostructures and are remarkable nanostructured photocatalysts, in large part due to their exceptional electron transport capabilities [3,23,24]. While nanostructured TiO_2_ possesses remarkable properties, it is not without its limitations. As a semiconductor, it exhibits a forbidden energy band gap where electronic transitions take place [25]. This band gap restricts TiO_2_ to respond only to UV light irradiation. The key challenge in TiO_2_ studies lies in progressively reducing the energy of the forbidden band to enable irradiation with visible light from the solar spectrum. Self-doping allows TiO_2_ to have its electrical characteristics modified. The introduction of Ti^3+^, oxygen vacancies, and surface disorders to TiO_2_ have all been linked to cathodization process. The resulting blue TiO_2_ has a narrower band gap [26,27,28]. When about dopants, they may be interstitial, substitutional, or both in the event of incorporation, all of which influence its action. Titanium dioxide changes its characteristics in response to its environment. It is challenging to compare the efficiency of different deposition procedures (sol-gel, solid reaction/mechanical activation, chemical vapor deposition, etc.) that use different precursors of metal ions in photocatalytic degradation or synthesis of organic molecules [29,30,31].

TiO_2_ properties can be improved with a variety of elements, including metals and non-metals [32,33] or by depositing [34] structures/elements in order to create a composite with special photocatalytic properties. Ions of transition metals are by far the most common. These elements are of particular interest because of the partial filling of their d-orbitals. When incorporated into the titanium dioxide lattice, new energy levels arise close to the conduction band [35,36]. Numerous has been undertaken to improve the photocatalytic activity of TiO_2_ with a transition metal oxide such as Fe_2_O_3_, ZnO, CuO, NiO, Co_3_O_4_, and V_2_O_5_ [37,38]. The presence of these metals efficiently enhances the properties of TiO_2_, resulting in a reduction in the band gap for photo-excitation (known as red shift) and a decrease in the recombination rate of photogenerated electron-hole pairs [37]. Despite the extensive exploration of various dopants, a significant focus has been placed on investigating Co- deposition on TiO_2_ thin films. These films have attracted considerable attention due to their ability to display ferromagnetic behaviour under typical room temperature conditions. This characteristic makes them highly suitable for many applications. Additionally Co-TiO_2_ thin films also show promise in enhancing their photocatalytic properties within the visible region, opening possibilities for efficient light-driven chemical reactions [39,40]. Several different techniques for Cobalt deposition on TiO_2_ have been detailed in the research literature. These include sol-gel dipping [41,42], plasma treatment [43,44,45,46], electrodeposition [47], electrochemical pulsed deposition [34,48], etc. 

Boutlala et al. [41] reported obtaining thin films of Cobalt-TiO_2_ that were produced using the sol-gel technique and deposited onto glass substrates, the predicted optical band gap decreases from 3.30 to 2.96 eV. Nana Liu et al. reported obtaining a Co-P/TiO_2_ composite through electrodeposition and with a rate of methyl orange degradation of about 13.3% [47]. In order to facilitate the photocatalytic degradation of methyl orange in water, Dalt et al. [49] create a nanotube/TiO_2_ composite and the degradation rate was almost 39.8%. In another paper [34], it was reported that the photoelectric catalysis process is enhanced by the porous Co(OH)_2_ nanoflakes coated upon TiO_2_ nanotubes through electrodeposition, which increases the specific surface area for light absorption and improves the contact between the electrode and electrolyte.

Therefore, based on our rigorous analysis of the existing literature, we conclude that the electrode used in this investigation has not been functionalized. When comparing our findings to those in the literature, we find that some dyes, such methyl orange, have been found to be degraded, although inefficiently, so here we report the degradation of doxycycline using a new catalyst with compromising degradation results.

In this study, we introduce a new photo-electro-catalyst designed for the DOX degradation from wastewater. Since the conductivity of TiO_2_ nanotubes can be improved by cathodization turning the color of TiO_2_ nanotubes from gray to blue as it was reported also in the literature [50], we propose two steps of fabrication process of the photo-electro-catalyst: blue TiO_2_ nanostructures are obtained through anodization followed by calcination, activation and cathodic polarization (cathodization) [51], which are then subjected to Cobalt deposition through either electrochemical techniques or plasma treatment. The resulting Cobalt-blue-TiO_2_ catalyst exhibits remarkable characteristics, including high interfacial charge transfer efficiency and slow recombination due to the presence of more localized defect states. The blue-nanotubes formation is verified by SEM, valence states by optical analysis, and the presence of Co is verified by EDX as the literature also reported [52,53].

Moreover, this study aims to emphasize the dual functionality of the photocatalyst obtained in our research. The photocatalyst possesses the unique ability to serve a dual purpose: firstly, it can effectively eliminate pathogenic bacteria present in water, contributing to water purification efforts. Secondly, it exhibits photo-electro-catalytic properties, enabling the efficient degradation and removal of organic compounds from water. By showcasing the photocatalyst’s versatile capabilities, this research highlights its potential in addressing both bacterial contamination and organic pollution in water sources. These nano-materials are attractive for future prospects because they may be utilized in a variety of applications, such as fuel cells, photocatalytic systems, and energy storage materials such as smart windows, in addition to sensors for gas and hydrogen production [54,55,56].

## 2. Experimental

### 2.1. Catalyst Preparation

#### 2.1.1. Blue TiO_2_ Nanostructures Electrochemically Obtained on Titanium Plates

Blue titanium dioxide nanostructures **(BTs)** were produced by anodizing recycled Titanium (Ti) plates taken from the aerospace industry (considered waste products from the production line) at 40 V for 2 h in a two-electrode cell. The precursor electrolyte is composed of ethylene glycol (EG) (Sigma Aldrich, St. Louis, MO, USA), 10% (vol.) of water, and 0.3% (vol.) of ammonium fluoride (NH_4_F) (Sigma Aldrich) (Solution 1). After anodization, the sample was submerged to calcination in an oven at 450 °C for 2 h. After this step, the sample is activated and more stabilized in the same anodization precursor solution for 10 min at 4 V. As a final step, a reduction is made in an EG solution that also contains H_2_O_2_ for 4 min at −40 V.

#### 2.1.2. Cobalt Deposition on the Blue TiO_2_ Nanostructures

To highlight the most efficient Cobalt (Co) deposition method on BTs previously obtained on titanium electrodes, two methods were applied: an electrochemical technique and plasma treatment. 

**Figure 1:**
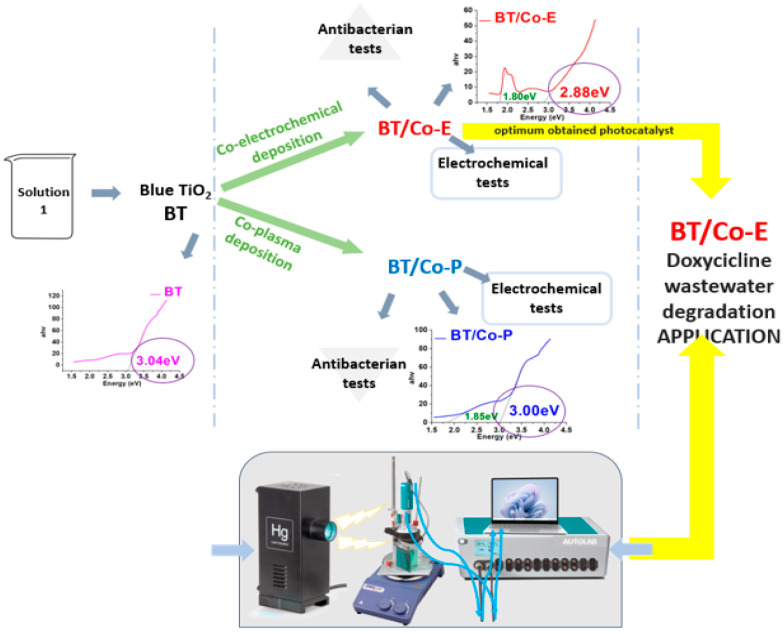
A description of the steps taken to create a photocatalyst with improved properties and an inset of the set-up—irradiation equipment used for irradiation.

The pulsed chronoamperometry method was used for the electrochemical Co deposition (see Figure 1). Electrochemical deposition is related to electrophoretic deposition, and takes place in an electrochemical cell, but unlike the electrophoretic process, electrodeposition involves a chemical bonding process. In electrochemical deposition, a solution is loaded into an electrochemical cell, and the substrate (in this case Blue-TiO_2_/Ti (BT)) acts as one of the electrodes [57]. When voltage is applied, the deposition conducting/semiconducting materials onto a substrate (often conducting) using an electric field, and a redox reaction takes place [58]. 

The method involves the use of a three-electrodes: BT samples previously obtained on Titanium plates are the working electrodes immersed into a specific Co solution; Ag/AgCl, KCl sat. and Pt mesh as the reference and counter electrodes, respectively. An Autolab (PGSTAT 302N) potentiostat/galvanostat from Metrohm (Herisau, Switzerland) system was used to control the parameters as well as collect data. Three steps were followed for Cobalt-blue-TiO_2_ **(BT/Co-E)** photocatalyst preparation: (i) applying −1.5 V for 2 s to initiate Co nucleation on BT substrate; (ii) maintaining open circuit potential for 5 s; (iii) followed by applying −0.95 V for 5 s to initiate Co deposition. Steps (ii) and (iii) were performed 200 times. 

According to the available research [45], an aqueous solution with a molar ratio of Co(NO_3_)_2_: HNO_3_ = 1:1 was used to obtain an aqueous solution of Co(NO_3_)_2_. An aqueous solution of hydrous Cobalt nitrate Co(NO_3_)_2_ ·6H_2_O that had been adjusted with HNO_3_ resulted in a solution that had a pH value of 4. As a result, a volume of solution containing Co(NO_3_)_2_ with a molar concentration of 0.05 M was utilized, and it was then adjusted using a volume of solution containing HNO_3_ with a concentration of 0.05 M.

The plasma Cobalt-Blue-TiO_2_ **(BT/Co-P)** sample was obtained by deposited Cobalt using plasma magnetron sputtering method onto BT plate support. We used a magnetron plasma source, with 1 inch Co target (99.9% from Kurt Lesker Company, Dresden, Germany) and RF power supply generator to generate plasma. Details about experimental plasma set-up descriptions are presented in our previous report [59]. In the present work, we use the following parameters: RF power supply of 100 W, a basic pressure at about 4 × 10^−5^ mbar (without gases), and a working pressure 5 × 10^−4^ mbar for 100 sccm of Ar. The deposition time of Co was 30 s. 

For an easy understanding of the work stages, the steps involved are shown in the Figure 1.

### 2.2. Catalyst Characterization and Applicability

To study the *morphological characteristics* of obtained catalysts, SEM and EDX were performed using Scanning Electron Microscope including Oxford EDX detector-analyser.

*Optical characteristics* were performed using Perkin Elmer (Turku, Finland) Lambda 650-850-950 UV-Vis spectrophotometer. 

*Wettability.* The sessile drop method was used along with an optical contact angle meter (Contact Angle Meter–KSV Instruments CAM 100 from Biolin Scientific, Västra Frölunda, Sweden) fitted with a camera to determine the contact angles of distilled water solvent onto samples.

*Electrochemical methods* as by Electrochemical Impedance Spectroscopy (EIS), Mott Schottky, and Cycle Voltammetry were performed to investigate the interfacial charge-separation efficiencies and stability of prepared photocatalysts, using potentiostat/galvanostat from the Metrohm Autolab (PGSTAT 302N) system. 

*The chemical composition* of the BT and BT/Co-E samples was analyzed by X-ray Photoelectron Spectroscopy (XPS). The XPS analysis was conducted using a K-Alpha Thermo Scientific (ESCALAB™ XI+, East Grinstead, UK) spectrometer equipped with a 180° double-focusing hemispherical analyzer. The peak positions were calibrated with respect to the standard C1s peak (284.8 eV). The surface elemental composition was determined by recording survey spectra at a pass energy of 50 eV. To evaluate the elemental bonding states of the as-investigated samples, high-resolution spectra for C1s, O1s, Ti2p, and Co2p binding energy regions were measured at a pass energy of 20 eV. Acquisition steps of 1 eV were used for the survey spectra and 0.1 eV for high-resolution spectra. The spectra acquisition and processing were performed using the advanced Avantage data software (Thermo Avantage v5 9921, East Grinstead, UK).

*Antibacterial activity*. The effectiveness of the materials’ antibacterial properties was measured against two different harmful microbial strains *Salmonella typhimurium*–ATCC 14028 and *Pseudomonas aeruginosa*–ATCC 15442. Luria Bertani Agar (LBA) plates were used for bacterial culture [60]. The LBA compositions are peptone (Merck, Rahway, NJ, USA), 10 g/L, yeast extract (Biolife, Layton, UT, USA) 5 g/L, NaCl (Sigma-Aldrich, St. Louis, MI, USA) 5 g/L, and agar (Fluka, Whitby, ON, Canada) 20 g/L. The stock of bacterial culture was kept at 4 °C.

Percentage of growth inhibition (I%) was used to assess antibacterial efficacy.

(1) $$I\%=\left[\left(B_{18}-B_{0}\right)-\left(C_{18}-C_{0}\right)\right]/\left(B_{18}-B_{0}\right)\times 100$$

where “I” is the rate of growth inhibition, B18 is the blank-compensated optical density (OD) at 600 nm, B_0_ is the blank-compensated OD_600_ of the positive control organism at 0 h, C_18_ is the negative control-compensated OD_600_ of the organism in the presence of test sample at 18 h, and C_0_ is the negative control-compensated OD_600_ of the organism in the presence of test sample at 0 h.

Sterilized samples were incubated in a Laboshake Gerhardt shaker for 18 h at 37 °C and 250 rpm in 10 mL of Luria–Bertani broth (the sterile medium was inoculated with 1% bacteria). To determine bacterial proliferation, the optical density of the samples and the control (bacteria culture without sample) was measured at 600 nm using a UV-VIS spectrophotometer (Jenway Spectrophotometer-Waltham, MA, USA).

*Photodegradation applicability.* DOX (hydrochloride salt, MW = 480.9 g mol^−1^) was purchased from DELOS Medica. As found in the literature [54], the antibiotic changes into different species were based on the pH. These species are DXCH^3±^ at pH 3, DXCH^2±^ at pH 6, and DXCH^−^ at pH 9. Moreover, 70% of the molecule at pH 3 is DXCH^3+^, 98% of the molecule at pH 6 is DXCH^2±^, and 75% of the molecule at pH 9 is DXCH^−^ [61].

DOX photodegradation was carried out in a quartz cell enclosed in a faradaic cage where outside light could not penetrate. Mercury vapor lamp used for the photodegradation process works at 30 W and generates a white light [62,63] (the lamp is fitted with filters from 200–600 nm wavelengths), illuminating the sample from one side of the reaction cell at around 5 cm. A D Lab MS-PA magnetic stirrer was used to control the stirring of the fluid. With NaOH and/or HCl solutions, the solution’s pH was adjusted to the desired value. Prior to irradiation, 10 mL of a known concentration DOX solution was added in the cell, and the stirring was turned on. Before being exposed to light, the photocatalyst electrodes were immersed in DOX solution for 30 min to achieve optimal DOX adsorption at the solid’s surface. A fixed volume of solution was exposed to light for various periods. 

All the characterizations and tests were run in triplicate for maximum reproducibility, averaging them, and calculating the standard deviation with the Excel function.

## 3. Results 

### 3.1. Characterization of the Synthesized Electrodes

#### 3.1.1. Physicochemical Characterization of BT, BT/Co-E, and BT-Co-P Electrodes by SEM, EDX, and Wettability

After the anodization process, TiO_2_ electrode is observed as white, but turns into a black color for 5 s after annealing at 450 °C, and after cathodic polarization it is stabilized to a blue color (obtained stable BT electrode) caused by the electrochromic effect [64].

**Figure 2:**
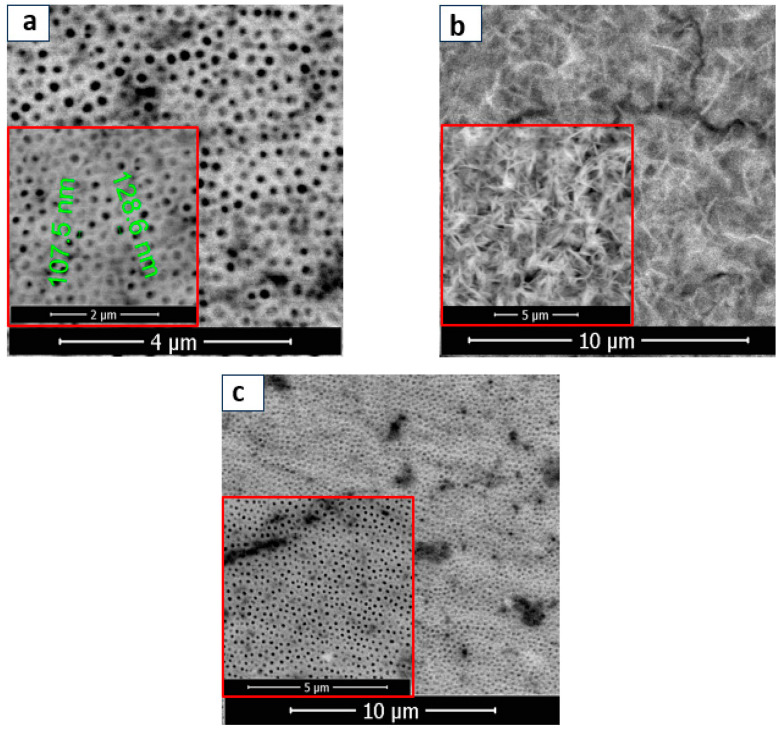
SEM images recorded at various magnifications for (**a**) BT; (**b**) BT/Co-E; (**c**) BT/Co-P.

In fact, the electrochromic effect is caused by the change from Ti^4+^ to Ti^3+^ and the intercalation of protons. The structure of BT film was well organized, and a thickening of the tubular walls is observed with the outer diameter of the nanotubes of 100 nm approximately (see Figure 2a). TiO_2_ is a 2-octahedron TiO_6_ base compound, with the Ti^4+^ ion in the centre surrounded by six oxygen ions [65]. According to the scientific literature [66], the loss of oxygen from the network releases free electrons. Self-doping occurs during the reduction process in EG solution with H_2_O_2_ for 4 min at −40 V, resulting in numerous structural defects in the TiO_2_ lattice that form shallow defect bands below the conduction band [67,68]. Thus, free electrons are either trapped in oxygen vacancies (VOs) to create color centers, which leads to the blue color of the TiO_2_ nanostructures or are captured by Ti^4+^ and form the Ti^3+^-VO-Ti^3+^ defect complex, which maintains charge neutrality. Exciton trapping is mostly associated with Ti^3+^ and oxygen defects in TiO_2_ with numerous structural defects.

**Figure 3:**
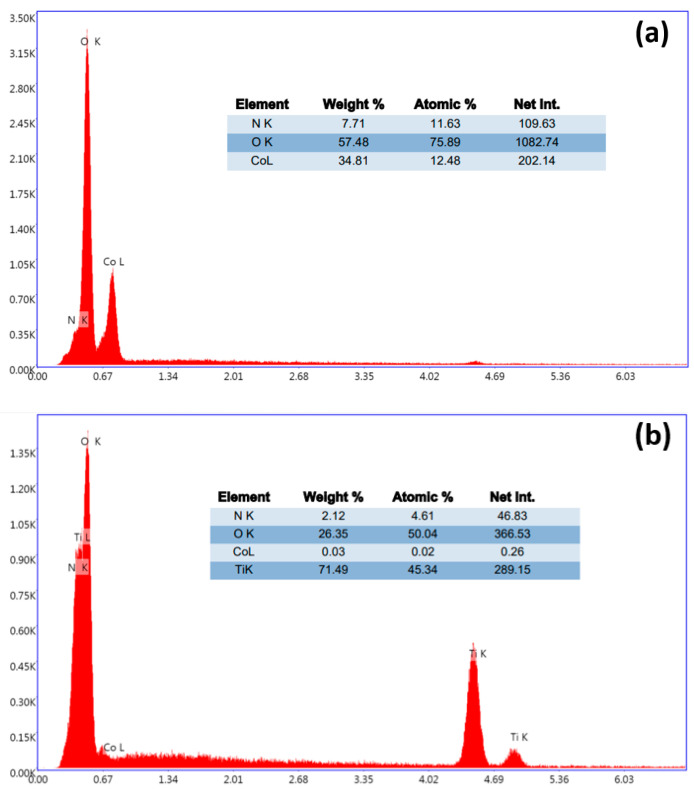
EDX spectra for (**a**) BT/Co-E and (**b**) BT/Co-P.

Following the metal deposition, SEM images were captured at different magnifications to examine various structures. The presence of Cobalt on BTs is highlighted by the formation of star-shaped nanostructures (BT/Co-E), according to Figure 2b. The BT surface was coated in a uniform layer of Co that was electrochemically deposited. EDX analysis (Figure 3a) confirms the presence of Cobalt in the BT nanostructure, revealing a weight of about 34.81%. 

Furthermore, Co deposition on BT nanostructures was accomplished using plasma technique. As a result, the SEM images for the BT/Co-P sample are displayed below (Figure 2c). In contrast to the previously discussed sample (BT/Co-E), there is no apparition of the formation of Co star-shaped structures. After Co deposition has been completed by plasma, there is no noticeable morphological change within the BT substrate (see Figure 2a,c). 

Also, the EDX results for the BT/Co-P sample showed a small amount of Cobalt (Figure 3b) of about 0.03%.

Contact angle analysis was used to investigate the wettability property of the samples. The outcomes are shown in Table 1, and it can be observed that BT/Co-E had a minimal contact angle. As the data show, Co presence was shown to be more effective at decreasing the contact angle. The hydrophilicity of these photocatalysts was confirmed by reducing the contact angle of surfaces with Co-Blue TiO_2_ compared to BT, which is an important metric for antibacterial behaviour. The higher the antibacterial action, the lower the contact angle [40].

#### 3.1.2. Optical Parameters—Band Gap Energy and Urbach Energy

As the literature reported, TiO_2’_s high band gap (approximately 3.2 eV in the anatase phase) limits its absorption to the solar spectrum’s ultraviolet rays, making titania powders unsuitable for use as a visible light absorber [69].

**Figure 4:**
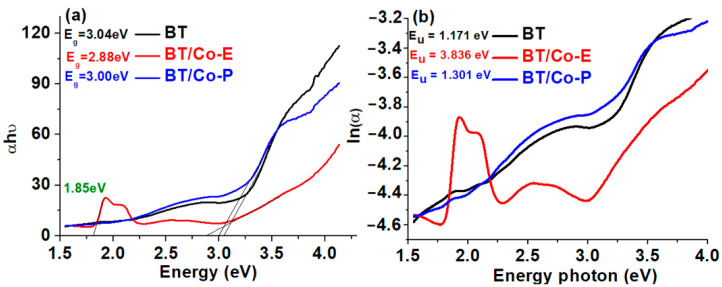
(**a**) Represented band-gap energy found from Tauc plot; and (**b**) ln(α) versus photon energy plots for Urbach energy determination.

As a result, the goal is to reduce the band gap, which in this case is reduced from 3.04 eV for BTs to 2.88 eV and 3 eV for BT films Cobalt modified with deposited Cobalt via electrochemical and plasma methods, respectively (Figure 4a). This is critical for creating visible light photocatalysis and studying other useful applications. The BT/Co-E sample shows a decrease in absorption in the UV region and an increase in absorption in the visible region in comparison to BT and BT/Co-P samples. The broad absorption in the visible region in BT/Co-E sample is due to ligand field transition of Co^2+^ in octahedral coordination [70], these results also are sustained by other studies [65] where the substitution of Co^2+^/Co^3+^ on the Ti^4+^ site was explained. When Co ions are electrochemically inserted into the TiO_2_ lattice, repulsive interactions between the Co^2+^ ions and the surrounding oxygen ions occur, separating the d-band states of Co^2+^ into ground and excited states, resulting in a d–d electronic transition in the visible region [65].

TiO_2’_s band gap was calculated by Tauc plot representing αhν vs. energy [71], were *E_g_* is the band-gap energy, *hν* is the energy of the incident photon and α is the absorption coefficient [72]. The introduction of some localized defect states into the band gap of Blue TiO_2_ by Cobalt deposition process is what causes the reduction in band gap in the case of BT/Co-E and BT/Co-P catalysts. Because of the very low concentration of Cobalt in the TiO_2_ network, as shown by the EXD data, the bandgap in the BT/Co-P sample is extremely close to BT, making it difficult to influence other associated properties. The BT/Co-E sample, on the other hand, had a lower band gap value due to a greater amount of Cobalt injected into the TiO_2_ lattice, which resulted in an increased amount of localized defect states associated with surface defect bands below the conduction band [67,68].

The Urbach tail, with an associated Urbach energy, is responsible for the absorption tail produced by these localized defect states, which extends far into the band gap [66]. The Urbach energy was calculated from the absorption spectrum using the formulas below [73]:

(2) $$\alpha =\alpha_{0}exp\left(\frac{h\upsilon }{E_{u}}\right)\;\mathrm{and}\;E_{u\;}={\left(\frac{d[\alpha h\upsilon ]}{d[h\upsilon ]}\right)}^{-1}$$

Here, α_0_ is a constant and E_u_ is the Urbach energy. 

The Urbach energy was calculated as E_u_ = 1/Slope, which is the inverse of the linear region beneath the band gap slope, from the graph ln(α) = f (photon energy) (Figure 4b). It is evident from the above that properties of the developed BT nanostructures electrochemically modified with Cobalt exhibit the performance features required for further photocatalysis applications. As shown in Figure 4b, the Urbach energy increased from 1.171 eV to 3.836 eV, indicating that BT/Co-E catalyst has more structural defects than BT. According to the EDX results (Figure 3a), a higher concentration of Co was loaded using an electrochemical method, increasing the light absorption spectral range of BT/Co-E catalyst to the visible region, and reducing the band gap, which is beneficial for improving photocatalytic performance [65].

#### 3.1.3. Antibacterial Activity

Co deposited on blue TiO_2_ nanostructures was tested for its antibacterial activity against *Salmonella typhimurium* and *Pseudomonas aeruginosa*. TiO_2_ nanostructures are well known for their photocatalytic activity, which can be utilized for antibacterial applications. The addition of metal ions or metal nanoparticles, such as Cobalt, can further enhance the photocatalytic and antibacterial properties of TiO_2_ nanostructures [74]. Depositing Co nanoparticles onto the surface of TiO_2_ nanostructures can lead to enhanced photocatalytic properties. These enhanced properties can promote the generation of reactive oxygen species (ROS) when exposed to light, which can destroy bacterial cell membranes and inactivate the bacteria [75]. 

**Figure 5:**
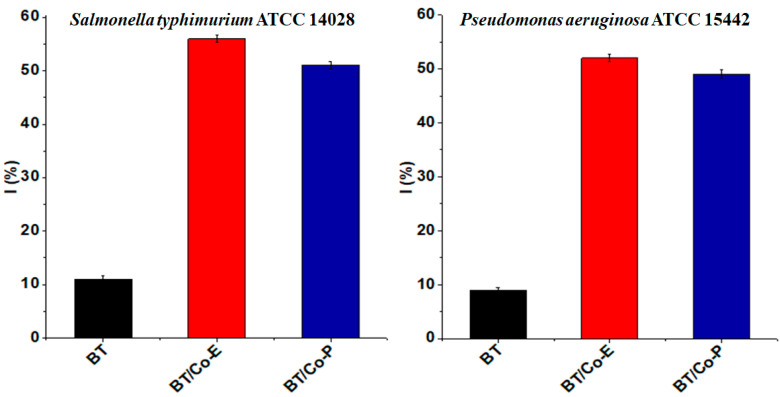
Antibacterial efficiency charts of the obtained samples against *Salmonella typhimurium* and *Pseudomonas aeruginosa*.

Figure 5 shows the antibacterial effect of the tested samples against the before mentioned bacteria. The antibacterial tests indicate that Co deposited on blue TiO_2_ nanostructures are highly effective against both Gram-negative and Gram-positive bacteria compared to a BT sample. A possible mechanism would be explained by the fact that when Co-deposited on TiO_2_ is exposed to light, it can generate electron-hole pairs. These can subsequently produce ROS-like hydroxyl radicals and superoxide anions. These ROS can attack bacterial cells, damage the cell membrane, and even interfere with cellular functions, leading to bacterial death. The “blue TiO_2_” usually indicates a phase of TiO_2_ with oxygen vacancies, which imparts a blue color. Oxygen vacancies can improve the charge carrier separation and enhance the photocatalytic activity. In combination with Co deposition, blue TiO_2_ can provide even better photocatalytic and antibacterial performance than regular TiO_2_.

According to the contact angle results (Table 1) all samples are hydrophilic. Bacteria tend to adhere more to hydrophilic (high wettability) surfaces. This is often attributed to the increased surface energy and the capability of such surfaces to form hydrogen bonds with bacterial cells. While bacteria might adhere more to hydrophilic surfaces, these surfaces can also facilitate easier removal of bacteria when water flows over them, especially if the adhesive forces between the bacteria and the surface are not too strong. Surfaces can be functionalized not just to control wettability but also to introduce antibacterial properties. For instance, surfaces can be coated with antibacterial agents that can kill or inhibit bacterial growth upon contact. The effectiveness of such agents might be influenced by the wettability of the surface. A surface’s wettability can also affect how bacteria encounter photocatalytic materials. Hydrophilic surfaces might allow bacteria to spread out and maximize contact with photocatalysts, enhancing the rate of bacterial eradication when exposed to light.

By designing surfaces where bacteria can easily attach and subsequently be removed, water purification systems can be made more effective. The antibacterial activity of Co-deposited TiO_2_ can be employed in various fields such as water disinfection, air purification, self-cleaning surfaces, and antibacterial coatings for medical devices [75]. 

The antibacterial performance of Co-deposited on blue TiO_2_ nanostructures might vary based on factors such as Co concentration, preparation method, light source, and exposure time. Optimizing these factors can maximize the antibacterial efficacy of the nanostructures [76].

#### 3.1.4. Electrochemical Features of the Developed Electrodes

**Figure 6:**
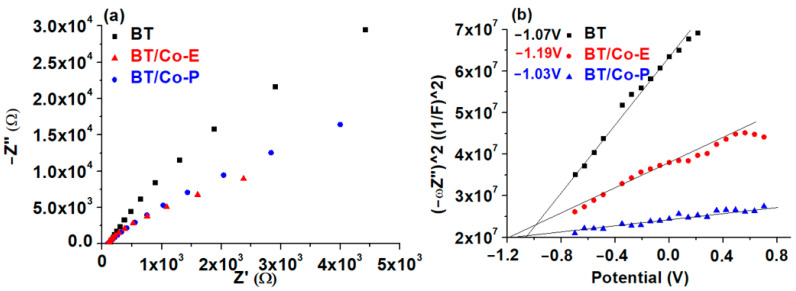
(**a**) Nyquist plots and (**b**) MS plots.

Electrochemical impedance spectroscopy (EIS) and Mott Schottky (MS) were used to estimate the charge transfer and recombination process at the electrode/electrolyte interface [77]. The EIS measurements for BT, BT/Co-E, and BT/Co-P are carried out at free potential in 0.9% NaCl solution and in the frequency domain between 0.01 and 10,000 Hz with an amplitude of ±10 mV. The Nyquist plots for these measurements are shown in Figure 6a. A Randles equivalent circuit, depicted in the inset of Figure 6a, was proposed to understand electrode behaviour by associating the arc radius with the charge transfer resistance (R_ct_) at the obtained catalyst/NaCl solution interface, which is in parallel with a constant phase element (CPE). R_ct_ values for BT, BT/Co-E and BT/Co-P were determined as 370 kΩ, 76 kΩ, and 143 kΩ, respectively. The BT/Co-E film with the lowest R_ct_ value has the highest interfacial charge transfer efficiency and the slowest recombination rate, which is an important factor to consider in catalytic degradation applicability.

The impedance data was analyzed using the Mott-Schottky Equation (3), which is defined as follows:

(3) $$\frac{1}{C^{2}}=\left(\frac{2}{q\varepsilon \varepsilon_{0N_{D}}}\right)\left(E-E_{fb}-\frac{kT}{q}\right)$$

where C is capacity of the space charge layer, q is the elementary charge, ε_0_ is the vacuum permittivity, ε is the dielectric constant, N_D_ is the concentration of donors, E is the applied external bias, E_f_ is the flat band potential, k is the Boltzmann’s constant, and T is the absolute temperature.

The slope of the Mott-Schottky plot reveals the donor concentration (N_D_), and the flat band potential (E_fb_), which is calculated by extrapolating to 1/C^2^ = 0, and is dependent on the recombination rate and interfacial charge transfer [21]. In contrast to normal TiO_2_, which has a positive slope [78], all Mott-Schottky plots for the obtained photocatalysts have a negative slope [79].

The calculated E_fb_ for BTs is −1.07 V and −1.03 V for BT/Co-P, which are close values due to a small amount of Co loaded by the plasma method, according to the EDX results and Urbach energy. In the case of BT/Co-E obtained through electrochemical deposition of BTs with Cobalt, E_fb_ is shifted to a more negative potential of −1.19 V, due to more localized defect states associated with higher Urbach energy. As a result, the electrochemical deposition method improves the photocatalytic abilities of the BT/Co-E surface, implying a slower recombination rate, which is in accordance with the decrease in charge transfer resistance determined by EIS [80].

The flat band potential values obtained from Mott-Schottky plots are commonly regarded as the conduction band potential (CB) for semiconductors [78].

Correlating with the variations in Urbach energy (Figure 6b) is a change in the donor concentration (N_D_) of BTs, BT/Co-E, and BT/Co-P from 1.32 × 10^21^ cm^3^ to 1.23 × 10^22^ cm^3^ and 2.77 × 10^21^ cm^3^. After electrochemical deposition with a greater quantity of Cobalt (according to EDX results), the flat band potential of BT shifts to more negative values. This suggests that the BT/Co-E film has better photocatalytic properties, because of improved charge transfer efficiency in bulk and at the electrode/electrolyte interface [79].

Finally, the BT/Co-E catalyst obtained through electrochemical Co deposition on BTs has the highest interfacial charge transfer efficiency and the slowest recombination rate, as well as better n-type conductivity than the BT/Co-P catalyst obtained through plasma magnetron sputtering. These properties are due to the presence of more localized defect states caused by a higher amount of Cobalt star-shaped nanostructures electrochemically deposited on BTs, which increases absorption at longer wavelengths and decreases the bandgap while also improving wettability and increasing antibacterial effect.

**Figure 7:**
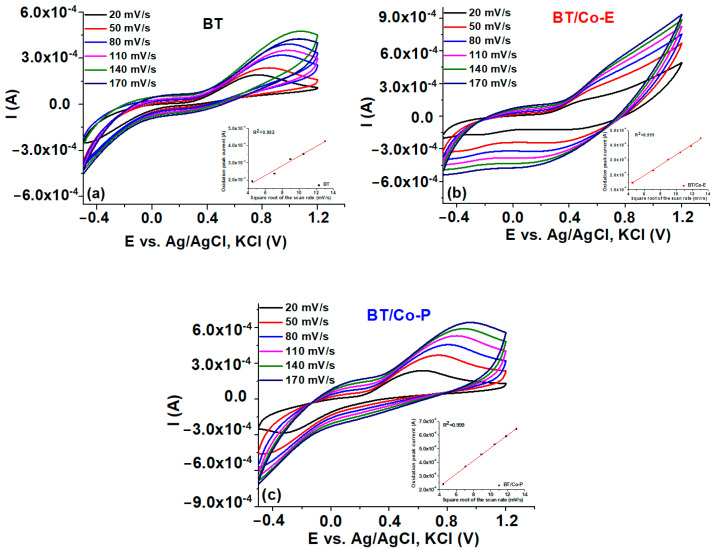
Cyclic voltammetry diagrams at various scan rates, with an inset displaying the plot of oxidation peak current densities vs. square root of potential scan rate.

Cyclic voltammetry (CV). The electrochemical behaviour of the electrodes, BTs, BT/Co-E, and BT/Co-P is studied in redox couple Fe^2+/^Fe^3+^ solution by cyclic voltammetry, at different scan rates of 20, 50, 80, 110, and 170 mV s^−1^, respectively. The anodic peaks have the same tendency, and it is clear that BT/Co-E exhibits better conductivity properties due to the higher current. The optimum anodic peak that corresponds to the oxidation process was located using these curves, and the corresponding diagrams were developed in the inset of Figure 7.

The cathodic peak of all samples is well defined and overlaps. The shift of the cathodic peak is observed to be in the negative direction, whereas the anodic peak exhibits a positive shift, with increasing scanning rates, indicates a desirable pseudocapacitive behavior for the utilization of these electrodes in catalytic applications [81]. Moreover, the capacitive behaviour of BT/Co-E electrode is more prominent, and current density increases, indicating a more conductive character than the BT and BT/Co-P electrodes. 

The electrolytes Fe^2+/^Fe^3+^ are crucial in maintaining the electro neutrality of redox processes. 

An enhanced redox peak is evident at voltages over 0.4 V across all samples. A notable enhancement in the electrochemical response of the BT/Co-E sample was noticed by a comparison of the CV curves of the electrodes before to and after surface functionalization. In the case of BT/Co-E, notable signals were seen in the cyclic voltammetry (CV) curves (Figure 7b), exhibiting significantly elevated current values with reduced onset potentials in comparison to both BTs and BT/Co-P. It is noteworthy to notice that BT/Co-E demonstrated superior redox peaks, potentially attributable to the greater Co content detected from the EDX data.

Straight lines are obtained when the peak value of the oxidation current is plotted against the square root of the scan speed for all studied electrodes (Inset Figure 7), indicating that the redox reaction is rapid and that ions diffusion in the obtained nanostructure controls the rate-determining step of the redox reaction. Due to the Co star-shaped structures electrochemically obtained on the BT nanostructure, according to SEM images (Figure 2b), the diffusion and charge transfer process of ions may be facilitated, which may explain why greater charge densities were reported for the BT/Co-E electrode.

#### 3.1.5. Proposed Energy Band Levels

Figure S1 depicts the schematic energy-level diagram for the investigated catalysts based on the MS E_fb_ results and UV-VIS E_g_ data. The measured CB and E_g_ were used to calculate the valence band (VB) energies. 

The BT/Co-P catalyst has the same VB value (1.97 eV) as BTs, indicating that plasma method does not significantly change the catalytic properties of BTs, as previously shown by surface and electrochemical data. BT/Co-E, on the other hand, has the narrowest bandgap energy of all the catalysts studied and VB is reduced by 0.62 eV, indicating that Co has been loaded through electrodeposition in an adequate amount and that the structure, optical, and electrochemical characteristics have been enhanced.

This makes the BT/Co-E catalyst the best candidate among all investigated catalysts for use in DOX photodegradation.

#### 3.1.6. Electrochemical Stability

**Figure 8:**
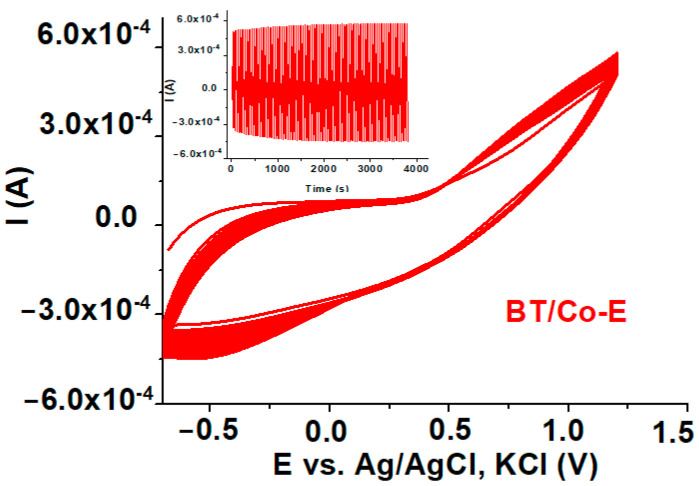
100 Cycle Voltammetry’s for BT/Co-E electrode in Fe^+3^/Fe^+2^ aqueous solution.

In order to establish the electrochemical stability of the Co electrochemically deposited on BT electrodes, 100 CVs cycles were recorded between −0.6 V to 1.2 V vs. Ag/AgCl at 100 mV s^−1^, in 0.5 M Fe^+3^/Fe^+2^ aqueous solution [82]. The results are illustrated in Figure 8. The associated current values remained practically constant as the number of CV cycles increased (inset of Figure 8), showing the strong binding of the electrochemically formed Cobalt coating on the BT substrate and good electrochemical stability.

The better the Cobalt adheres to the BT substrate, the higher the electrochemical stability of the electrode, and Figure 8 shows stable and good capacitive behaviour for the BT/Co-E electrode, with all 100 CV cycles practically overlapping.

#### 3.1.7. X-ray Photoelectron Spectroscopy (XPS)

Understanding the chemical structure of modified titania is crucial in comprehending the performance of TiO_2_ nanotubes and catalysts derived from them. X-ray photoelectron spectroscopy (XPS) is an appropriate technique for examining the chemical structure of these materials. The purpose of this study is to analyze the chemical composition of BT and BT/Co-E samples and to compare the surface environment of the TiO_2_ nanotubes before and after the reaction. XPS analysis was conducted on the BT and BT/Co-E samples to assess any alterations in their surface environment. Upon analysis, it was found that Cobalt was present on the surface of the BT/Co-E sample, as confirmed through EDX analysis, while titanium was notably absent. These findings imply that the star-shaped structure made of Cobalt, which was deposited on the surface of the TiO_2_ nanotubes, has effectively covered the entire surface of the blue nanotubes. 

**Figure 9:**
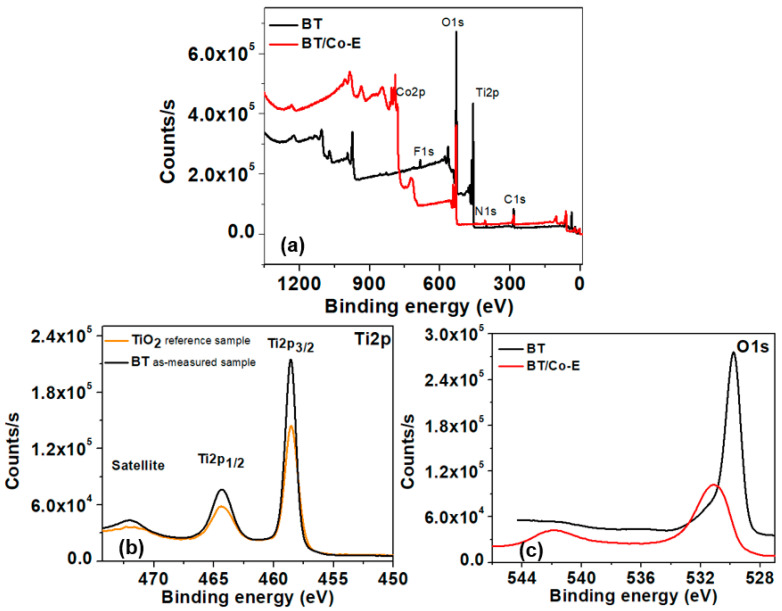
(**a**) Survey analysis of BTs, BT/Co-E and XPS spectra for (**b**) Ti2p and (**c**) O1s.

Figure 9 displays XPS spectra of high-resolution scans conducted in the Ti2p and O1s range for blue TiO_2_ nanotubes and Cobalt-deposited blue TiO_2_ nanotube catalysts. The distinctive peaks at 458.57 and 464.25 eV in the Ti2p spectrum of BTs confirmed titanium’s oxidation state to be +4, with a spin-orbit splitting of ΔE = Ti2p_3/2_ − 2p_1/2_ of 5.68 eV. Similarly, the O1s spectra of BTs showed the distinctive peaks of Ti-O connections at 529.82 eV. The BT/Co-E catalyst exhibits binding energy values of 531.20 eV and 542.06 eV for O1s, which can be attributed to the formation of Ti-O-Co bonds [83]. These results suggest that the chemical composition of the BT/Co-E catalyst is distinct from that of the BT sample, underscoring the importance of understanding its unique structure in relation to its catalytic performance.

### 3.2. Applicability of the BT/Co-E Catalyst

**Figure 10:**
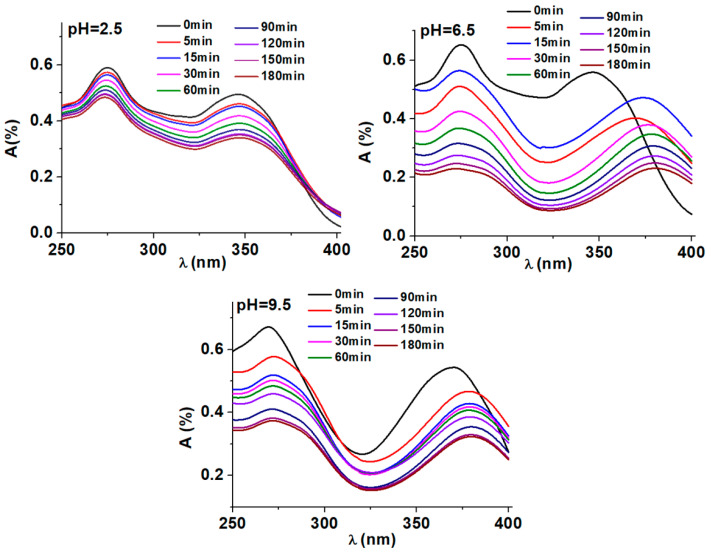
Evolution of UV-Vis spectra in the DOX solution during varied reaction times in pH-controlled photodegradation experiments.

*Influence of pH on the photocatalytic degradation of DOX.* Figure 10 displays the variations in UV-Vis spectra of a DOX solution throughout a standard photodegradation experiment carried out at different pH values, 2.5, 6.5, and 9.5. In presence of BT/Co-E catalyst, the DOX solution shows a spectrum with two maxima observed at 271 nm and 375 nm [61]. Depending on the chosen pH, the DOX solution shows different colors. At an acidic pH no coloration of the solution is observed, at a pH of 6.5 the solution becomes slightly yellow and at a pH of 9.5 the solution becomes slightly pink, as it was observed also in other studies from the literature [61].

It is significant to note that even after many hours of exposure, the absorbance at any wavelength never stabilizes, indicating that complete photo-degradation was not accomplished. Under the conditions of our experiment, the findings indicate that the photodegradation of DOX is quite distant from reaching equilibrium. Furthermore, these findings reveal that photodegradation and the mechanism are very pH dependent. Better results when exposing the catalyst to light are observed at a pH of 6.5, so compared to the other degradations at the other pHs, the absorption reduces significantly. 

*Photo-electro-catalytic application.* Going forward with our studies, it may be deduced that the best outcomes are obtained at a pH of 6.5. As a result, for further testing, a 6 mol/L DOX solution with a pH of 6.5 will be utilized. Both photodegradation and photoelectrodegradation tests were conducted to create a dual system in which DOX degradation from water occurs. An efficient and cost-effective system is characterized by the synergic combination between light and a photocatalyst to generate reactive oxygen species. 

**Figure 11:**
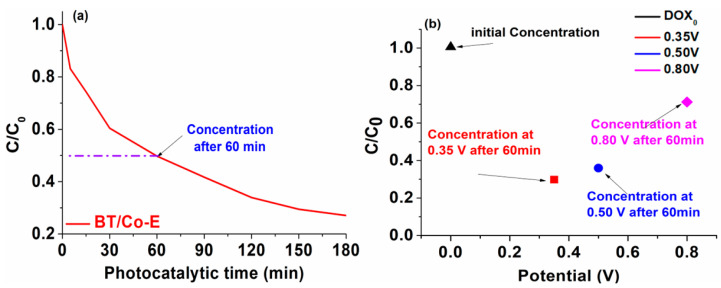
(**a**) Photocatalytic and (**b**) photo-electro-catalytic DOX degradation efficiency.

The efficiency of photodegradation and photoelectrodegradation will be compared here. The DOX solution was photoelectrocatalytically degraded at three distinct potentials, 0.35 V, 0.50 V, and 0.80 V, to demonstrate the impact of input potential on DOX degradation efficiency. According to recent research in the literature [84], the oxidation potential of TCs is founded at almost 0.80 V, although as shown in the data below (Figure 11b), the degradation of DOX has a poor response to this potential. The oxidation potential value of DOX was drastically reduced to 0.35 V due to BT/Co-E with enhanced catalytic features, so this is why three additional imposed potentials were chosen. 

After only 60 min, the photo-electro-catalytic system reaches 70% degradation efficiency, as can be seen in Figure 11b. An efficiency of 50% degradation is attained after 60 min in the photocatalytic system (Figure 11a). Given that the photocatalysis investigations were conducted over a period of 180 min, it was found that the degradation reached 80% efficiency, but only after irradiating for a triple time compared to the time allocated to the photoelectrodegradation.

It was clearly observed that the system does not work as well only when exposed to UV-Vis light, so the system will be coupled up to a potentiostat once the 60 min of photodegradation process is done. This potentiostat will apply a potential of 0.35 V, effectively quickly eliminating the compounds that have resulted from the photocatalytic degradation and which were not previously degraded. This could be because when exposed to light, the electron-hole pairs generated by the photons were quickly separated. This separation resulted in a significant number of electrons being produced, which facilitated the transfer of electrons through the electrode. As a result, the photocurrent, which is the current generated by light, showed a rapid improvement upon the onset of light exposure. Eventually, the photocurrent reached a stable state after continuous exposure to light.

The degradation kinetics, was calculated for the photocatalytic degradation using the Langmuir–Hinshelwood pseudo first-order kinetic model [85], which is shown in the equation below, was used to study the speed of the process.

(4) $$Ln\left(\frac{C}{C_{0}}\right)=k\times t$$

where C_0_ and C are the initial concentration (mol/L) and final concentration (mol/L) of DOX and t (min) and k (min^−1^) are the solution concentration and the rate constant.

Figure S2 shows that for DOX degradation in the presence of BT/Co-E catalyst and UV-Vis light, the relationship between $Ln\left(\frac{C}{C_{0}}\right)$ time (min) represents a logarithmic exponential growth. The R^2^ values for the association coefficients were greater than 0.900, and the first-order kinetic model did a good job of fitting the experimental data. Looking closely at the degradation diagram, two distinct degradation tendencies can be seen [86]. Based on the slope of the plots, the first-order rate constants (k) were found to be 15 × 10^−3^ min^−1^ for the first degradation tendency (0–30 min) and 0.5 × 10^−2^ min^−1^ (30–180 min) for the second degradation tendency [87]. 

#### Proposed Mechanism of DOX Degradation in the Presence of BT/Co-E

**Figure 12:**
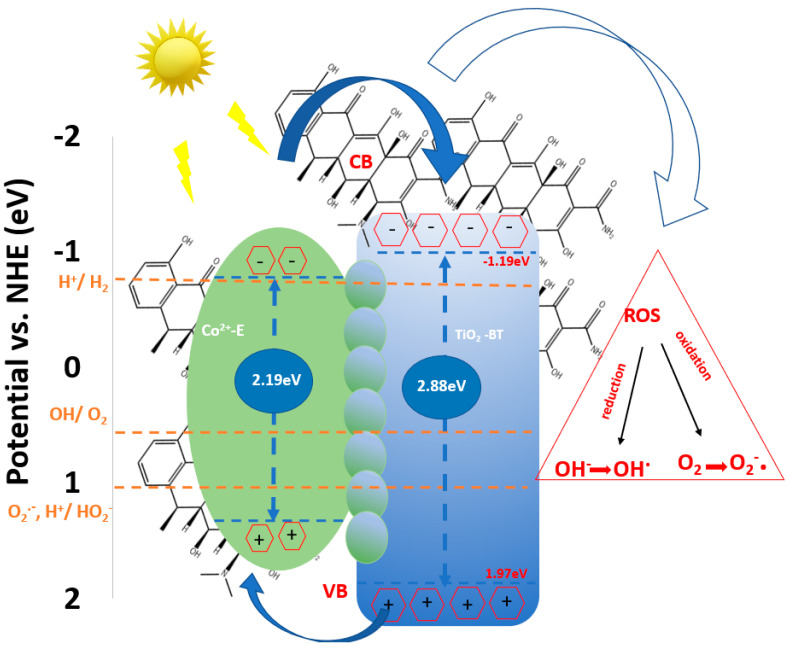
The mechanism of BT/Co-E photocatalyst.

Based on the results of the characterisation and photocatalytic activity and based on the other literature reports [54,88], the likely mechanism of BT/Co-E for DOX degradation is shown in Figure 12. It is possible that the following factors contribute to TiO_2_’s enhanced photocatalytic performance. 

Even though the conduction band (CB) level of TiO_2_ is more negative than the H^+^/H_2_ reduction, the fast exchange rate of CB electrons and valence band (VB) holes is what gives the TiO_2_ its photoelectrochemical activity. Cobalt ions could be added to the TiO_2_ nanotubes to make it work a lot better, which would lead to organic molecules photodegradation. A Ti-O-Co hybridization energy level is produced when exposed to visible light, resulting in narrowing the bandgap. This reduction enhances the ability to excite electrons [89]. As the catalytic process unfolds, a small quantity of dissolved O_2_ from wastewater reacts with the electrons generated by light, electrons excited from the Co-E CB that were caught and transported to TiO_2_, resulting in the production of a limited number of O_2_^−^· radicals. This process will improve the electron hole separation efficiency [22]. 

Furthermore, a limited quantity of holes generated by light, situated in the VB, engage with water to produce OH and OH^−^**·**. In addition, the holes present in the modified photocatalyst valence band (VB) will be activated upon exposure to light, leading to the conversion of water into hydroxyl-type compounds/hydroxyl radicals that will produce photocatalytic degradation.

## 4. Conclusions

The main findings of this study consist of performing new stable photo-electro-catalyst with antibacterial properties, based on reduced TiO_2_ nanotubes (blue nanotubes) and Cobalt ions deposited on the surface through electrochemical pulsed deposition and plasma magnetron sputtering, respectively.

The results showed that the star-shaped Cobalt-Blue-TiO_2_ film obtained by an electrochemical pulsed technique (BT/Co-E) gives the best results to nanoporous Cobalt-Blue-TiO_2_ film obtained by plasma magnetron sputtering (BT/Co-P). This is due to its higher interfacial charge transfer efficiency and lower recombination rate.

Band gap reduction in the BT/Co-E film can also be attributed to the electrochemical deposition of a higher amount of Co (observed in EDX spectra), which increases the number of localized defect states in the TiO_2_ band gap. Because of this, Urbach energy increased from 1.171 to 3.836 eV while the band gap energy decreased from 3.04 to 2.88 eV making BT/Co-E material an effective catalyst for a variety of applications, including photocatalysis, photo-electrocatalysis, water splitting, solar cells, the development of smart windows, batteries, etc. Furthermore, the pseudocapacitive behaviour and very good electrochemical stability of the BT/Co-E electrode, as established by electrochemical studies, suggests that this electrode is suitable for use in technological applications requiring catalytic qualities. 

The obtained catalysts were also studied from an antimicrobial point of view. The results showed that the presence of Cobalt in both obtained films improves the antibacterial activity against *Salmonella* and *Pseudomonas aeruginosa*. The BT/Co-E composite film had the greatest antibacterial impact, owing to a higher amount of Co deposited on the blue TiO_2_ nanostructures by the pulsed electrochemical method, which also improved the wettability behaviour of the catalyst, indicating that this catalyst will eradicate antibiotic-resistant bacteria from wastewater.

All tests performed in this study, such as electrochemical, optical, and antibacterial, showed enhanced results for BT/Co-E catalyst in comparison with BT/Co-P. Thus, this sample was employed to photodegradation and photoelectrodegradation processes of the doxycycline antibiotic. In both cases, the effects of the degradation were satisfactory. Our findings shows that with the photocatalytic system, 50% DOX was degraded after 60 min, and 80% DOX was degraded after 180 min. After 60 min of using the photoelectrocatalytic irradiation device, 70% yield was reached. The study explores the synergistic combination of light and applied potential makes the suggested system extremely straightforward, efficient, cost effective, and suitable for many other applications. Since the proposed system is dual, it operates independently; however, in the future, we aim to investigate the efficacy of a combined system in which bacteria are present in the water alongside the organic pollutant and light irradiation produces both the degradation of the organic compound and bacterial inhibition. This could be a limitation. Another potential flaw with this investigation is that it may not take into account the compounds formed during after the photocatalysis process.

## Figures and Tables

**Table 1:** Contact angle measurements.

Sample	Contact Angle (°) Water Solvent
**BT**	30 ± 0.13
**BT/Co-P**	10 ± 0.04
**BT/Co-E**	6 ± 0.05

## Data Availability

The raw/processed data generated in this work are available upon request from the corresponding author.

## Supplementary Materials

The following supporting information can be downloaded at: https://www.mdpi.com/article/10.3390/ma16247509/s1.
