# Supplementary material for: Photocatalyst Based on Nanostructured TiO2 with Improved Photocatalytic and Antibacterial Properties
Source: Materials (Basel). 2023 Dec 5;16(24):7509. doi: 10.3390/ma16247509 (PMC10744369; doi:10.3390/ma16247509)
Supplement: Supplementary file 1 [file materials-16-07509-s001.zip › materials-2708946-supplementary.pdf]

Supplementary Materials

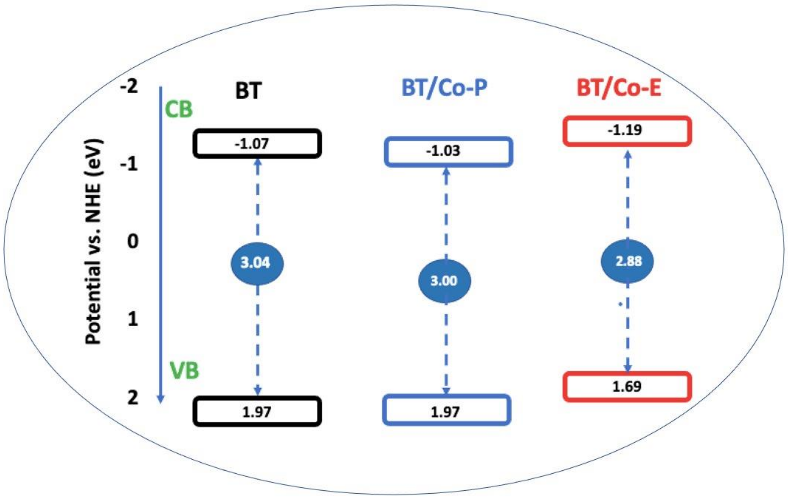

Figure S1. Schematic energy–level diagram of the BT, BT/Co-P and BT/Co-E photoelectrodes.

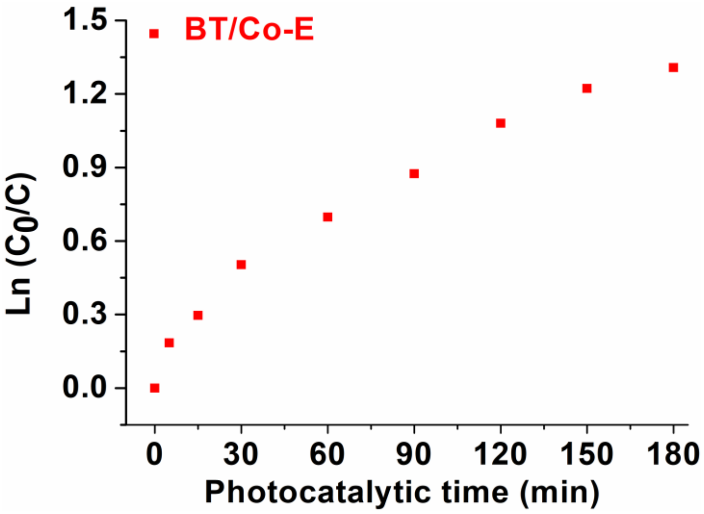

Figure S2. Photodegradation kinetics curve of DOX, pH=6.5
